# Development of a high density 600K SNP genotyping array for chicken

**DOI:** 10.1186/1471-2164-14-59

**Published:** 2013-01-28

**Authors:** Andreas Kranis, Almas A Gheyas, Clarissa Boschiero, Frances Turner, Le Yu, Sarah Smith, Richard Talbot, Ali Pirani, Fiona Brew, Pete Kaiser, Paul M Hocking, Mark Fife, Nigel Salmon, Janet Fulton, Tim M Strom, Georg Haberer, Steffen Weigend, Rudolf Preisinger, Mahmood Gholami, Saber Qanbari, Henner Simianer, Kellie A Watson, John A Woolliams, David W Burt

**Affiliations:** 1 Aviagen Ltd, Midlothian, UK; 2 The Roslin Institute, University of Edinburgh, Midlothian, UK; 3 Affymetrix, Santa Clara, CA, USA; 4 The Pirbright Institute, Compton Laboratory, Compton, UK; 5 Hy-Line International, Dallas Center, IA, USA; 6 Institute of Human Genetics, Helmholtz Zentrum, 85764, Neuherberg, Germany; 7 Institute of Bioinformatics and System Biology, Helmholtz Zentrum München, 85764, Neuherberg, Germany; 8 Institute of Farm Animal Genetics, Friedrich Loeffler Institut, Neustadt-Mariensee, Germany; 9 Lohmann Tierzucht GmbH, Cuxhaven, Germany; 10 Animal Breeding and Genetics Group, Georg-August-University Goettingen, Goettingen, Germany

**Keywords:** Genotyping array, Chicken, SNP

## Abstract

**Background:**

High density (HD) SNP genotyping arrays are an important tool for genetic analyses of animals and plants. Although the chicken is one of the most important farm animals, no HD array is yet available for high resolution genetic analysis of this species.

**Results:**

We report here the development of a 600 K Affymetrix® Axiom® HD genotyping array designed using SNPs segregating in a wide variety of chicken populations. In order to generate a large catalogue of segregating SNPs, we re-sequenced 243 chickens from 24 chicken lines derived from diverse sources (experimental, commercial broiler and layer lines) by pooling 10–15 samples within each line. About 139 million (M) putative SNPs were detected by mapping sequence reads to the new reference genome (*Gallus_gallus_4.0*) of which ~78 M appeared to be segregating in different lines. Using criteria such as high SNP-quality score, acceptable design scores predicting high conversion performance in the final array and uniformity of distribution across the genome, we selected ~1.8 M SNPs for validation through genotyping on an independent set of samples (n = 282). About 64% of the SNPs were polymorphic with high call rates (>98%), good cluster separation and stable Mendelian inheritance. Polymorphic SNPs were further analysed for their population characteristics and genomic effects. SNPs with extreme breach of Hardy-Weinberg equilibrium (*P* < 0.00001) were excluded from the panel. The final array, designed on the basis of these analyses, consists of 580,954 SNPs and includes 21,534 coding variants. SNPs were selected to achieve an essentially uniform distribution based on genetic map distance for both broiler and layer lines. Due to a lower extent of LD in broilers compared to layers, as reported in previous studies, the ratio of broiler and layer SNPs in the array was kept as 3:2. The final panel was shown to genotype a wide range of samples including broilers and layers with over 100 K to 450 K informative SNPs per line. A principal component analysis was used to demonstrate the ability of the array to detect the expected population structure which is an important pre-investigation step for many genome-wide analyses.

**Conclusions:**

This Affymetrix® Axiom® array is the first SNP genotyping array for chicken that has been made commercially available to the public as a product. This array is expected to find widespread usage both in research and commercial application such as in genomic selection, genome-wide association studies, selection signature analyses, fine mapping of QTLs and detection of copy number variants.

## Background

High density (HD) genotyping arrays have become extremely valuable tools for genomic analyses of human, model organisms and farm animal species due to their genome-wide coverage and high throughput nature. Such arrays are being widely used for many purposes including the detection of genetic associations with complex traits, fine mapping of quantitative trait loci (QTL), detecting signatures of selection, and increasingly for implementing genomic selection (GS) to farm animal species. In this paper, we describe the development of a HD genotyping array (600 K) for chicken, which is the first of this magnitude in chicken and has been commercially released for both public and proprietary use.

The chicken is not only a major livestock animal but also an excellent model organism for genetic and evolutionary studies such as for detecting natural and artificial selection under domestication [1-3]. A large amount of genomic information is already publicly available for this species including a near-complete reference genome, more than 3.5 M genetic variants in public databases (such as dbSNP), and more than 3,000 QTLs in the Chicken QTLdb (http://www.animalgenome.org/cgi-bin/QTLdb/GG/index). In spite of all these resources no HD genotyping array is yet available for this species that can facilitate high throughput investigation of many individuals for research and commercial breeding. A number of studies, however, have reported the use of low to medium (3 K to 60 K) density panels for various purposes [4-7] but these panels were proprietary and have not been made available for wider use. In contrast, for cattle, for example, two HD genotyping arrays (both >600 K) are now available [8,9].

A major determinant of the utility of an array is the density of its marker panel. Our goal in developing the HD array for chicken was that the array should be versatile and suitable for addressing the different purposes described above in a diverse range for chicken breeds and populations. The effectiveness of an array will also depend on its ability to exploit the linkage disequilibrium (LD) structure of the genome of the target population. Previous studies have shown that the LD pattern in chicken varies not only across chromosomes and genomic regions but also varies widely among breeds [10-13]. Moreover, previous studies [2,14] have also shown that a large amount of genetic variation still exists both within and among the domesticated and commercial chicken breeds despite many generations of selection. All these indicate that to achieve a wide applicability of the array for diverse breeds, an HD panel would be more desirable than low or medium density ones. For instance, according to Megens *et al.*[12] a whole-genome marker assay for chicken would require more than 100 K SNPs (single nucleotide polymorphism) to exploit the LD and haplotype information. Therefore to achieve this we set the target of including in the array as many SNPs as possible with an even coverage of the genome, limited only by the capacity of the genotyping platform (in this case the Affymetrix® Axiom® platform). These limits are determined by the allelic types of the variants and the number of probes required for querying those.

Developing a HD array, however, requires a large collection of SNPs segregating within populations to select from. Several studies have reported detection of a large number of SNPs from chicken, most notably, the detection of 2.8 M SNPs by Wong *et al.*[14] by analysing single birds from three breeds, and detection of over 7 M SNPs by Rubin *et al.*[2] by analysing pooled samples from each of eight domesticated broiler and layer lines. Nevertheless, for practical applications using a HD genotyping array we intended to create a larger catalogue of SNPs that are segregating within diverse chicken populations. Next generation sequencing (NGS) technology has made the whole-genome sequencing of many individuals practical by making it affordable and rapid. We, therefore, used a NGS platform (Illumina GAIIx) to re-sequence 243 birds from 24 lines representing commercial broiler and layer breeds, and several experimental and inbred lines. Similar to Rubin *et al.*[2] we also adopted a pooled DNA sequencing approach and aligned the sequenced reads to the chicken reference genome for detecting SNPs. Using standard criteria we detected ~139 M SNPs of which about 78 M were segregating within one or more lines. We used several stringent criteria to reduce the number to a set of high quality SNPs and then applied an iterative algorithm to select a list of ~1.8 M SNPs, evenly distributed across the genome, for further validation. We used the genetic map distance (cM) rather than physical distance (kb) to account for the differential recombination rates across the chromosomes to assess the evenness of distribution across the genome. The final array was created using 580,954 validated SNPs. This paper describes the detection, selection and validation of SNPs to create a 600 K HD Affymetrix® Axiom® genotyping array and discusses the potential application of this array.

## Results and discussion

### Sequencing of birds and mapping of sequence reads

The 243 sequenced individuals originated from 24 lines including four commercial broilers, six commercial white egg layers (WEL), five commercial brown egg layers (BEL), eight experimental inbred layers and one unselected layer line (further details about the lines are provided in the Table 1). Over 4.8 billion reads were generated by sequencing the samples. When aligned against the new chicken reference genome (*Gallus_gallus_4.0*, pre-published version), about 3.9 billion reads (~80%) mapped to the genome and about 3.6 billion reads (~74%) mapped with a mapping quality score ≥ 20. While the intended depth of coverage of sequencing was 10–20 folds, the actual coverage varied between 8 and 17 fold across different lines, with the potential loss appearing from failure of some reads to map at all or map with confidence (i.e. with the mapping quality score at least 20). Although the current build of the reference genome (*Gallus_gallus_4.0*) represents ~96% of the chicken genome, some of the smaller micro-chromosomes are not yet represented [15]. Besides chromosome 16 (Chr16) is only partially represented with the length of about 539 kb whereas the full estimated size of this chromosome is 9–11 Mb [16]. This is one of the reasons for failure of some reads to map to the genome, the other possible reasons being the presence of repeat contents and poor quality of some reads.

**Table 1:** Description of sequenced samples and the number of segregating SNPs detected from different lines

**Line**	**Breed**	**Usage**	**Samples**	**Analysis**	**Number of SNPs detected (with quality score ≥20)**
B1	Composite	C	10 ♂	Pooled	9,525,306
B2	Composite	C	10 ♂	Pooled	10,760,626
B3	Composite	C	10 ♂	Pooled	9,305,545
B4	Composite	C	10 ♂	Pooled	10,422,420
WEL1	White Leghorn	C	10 ♂	Pooled	8,605,672
WEL2	White Leghorn	C	10 ♀	Pooled	9,900,302
WEL3	White Leghorn	C	10 ♀	Pooled	7,340,262
WEL4	White Leghorn	C	10 ♂	Pooled	7,951,423
WEL5	White Leghorn	C	10 ♂	Pooled	11,139,255
WEL6	White Leghorn	C	3 ♀	Individual	2,630,343
BEL1	White Plymouth Rock	C	10 ♀	Pooled	8,182,562
BEL2	White Plymouth Rock	C	10 ♂	Pooled	7,876,558
BEL3	Rhode Island Red	C	10 ♂	Pooled	11,593,980
BEL4	Rhode Island Red	C	15 ♀	Pooled	3,236,749
BEL5	White Rock	C	15 ♀	Pooled	3,751,628
RI-J	Brown Leghorn	E	10 ♂	Pooled	10,358,702
I1	IAH Inbred 7_2_	E	10 ♀	Pooled	447,683
I2	IAH Inbred P	E	10 ♀	Pooled	1,816,948
I3	IAH Inbred Wellcome	E	10 ♀	Pooled	1,109,100
I4	IAH Inbred N	E	10 ♂	Pooled	1,055,257
I5	IAH Inbred 15 l	E	10 ♀	Pooled	801,052
I6	IAH Inbred 0	E	10 ♀	Pooled	1,395,679
I7	IAH Inbred 6_1_	E	10 ♀	Pooled	803,892
I8	IAH Inbred C	E	10 ♀	Pooled	603,338

(Abbreviations: B, Broiler; WEL, White egg layer; BEL, Brown egg layer; I, Inbred, IAH, Institute for Animal Health; RI, Roslin Institute; C, Commercial; E, Experimental).

To be able to detect SNPs from unmapped sequence regions, which potentially include unrepresented micro-chromosomes, we created a *de novo* assembly of the good quality unmapped reads. This resulted in 885,704 contigs with a total size of approximately 38 Mb. For detection of SNPs, we only used contigs with a length greater than 300 bases. There were 2,374 such contigs with the total length of about 1.1 Mb. The mean size of these contigs was about 457 bases with a maximum size of 7.8 kb.

### SNP detection

Initially SNP detection was done separately within each line. The criteria used for calling SNPs were: (1) the SNP position must be covered by at least five sequencing reads with a map-quality score ≥ 20; (2) relevant bases must have a base-quality score ≥ 20 and (3) the SNP quality score must be ≥ 20. To minimise the risk of detecting false positives from duplicated genome regions, any SNPs with coverage more than three standard deviations from the mean coverage were excluded. The number of segregating SNPs detected per line ranged between 448 K to over 11 M (Table 1) with average numbers being approximately 10 M, 7.7 M and 1 M for broiler, layer and inbred lines respectively. In total 78 M segregating SNPs were detected from this within-line analysis. Apart from these SNPs, an additional 5,069 SNPs were identified from the contigs that were assembled *de novo*. Additional file 1: Table S1 (in SupplementaryTableS1.xlsx) shows the number of SNPs and their proportional distribution across the chromosomes. Much fewer SNPs (only 52,974) were detected from Chr16 mainly because of the partial representation of this chromosome in the current reference genome. On the contrary, in spite of a very high quality assembly for chromosome Z (ChrZ) in the current reference genome, we observed a very low density of SNPs on this chromosome (only ~3 SNPs/kb compared to an average of ~78 SNPs/kb across the genome). Our result, however, corroborates with the findings of previous studies which reported reduced genetic variations on this chromosome for a multitude of potential reasons such as low male effective population size due to skewed reproductive success among males, selective sweep due to selection on sex-linked characters combined with lower recombination rates on ChrZ than on autosomes [17].

We investigated what proportion of the publicly available SNPs was also discovered in our project. On request, we received from NCBI, a list of about 11.7 M SNPs (the majority of which are yet to be updated in the current version of chicken dbSNP list). Comparison suggests, about 65% of these SNPs are present in our 78 M list.

In order to call SNPs from low coverage regions with greater confidence, we combined reads from all the 24 lines to perform a “global analysis”. The same SNP-calling criteria mentioned for the within-line analyses were used for this analysis resulting in the discovery of ~139 M SNPs. This analysis, however, detected not only segregating SNPs within lines but also the ones that are fixed in different lines for alternate alleles.

### SNP selection

**Figure 1:**
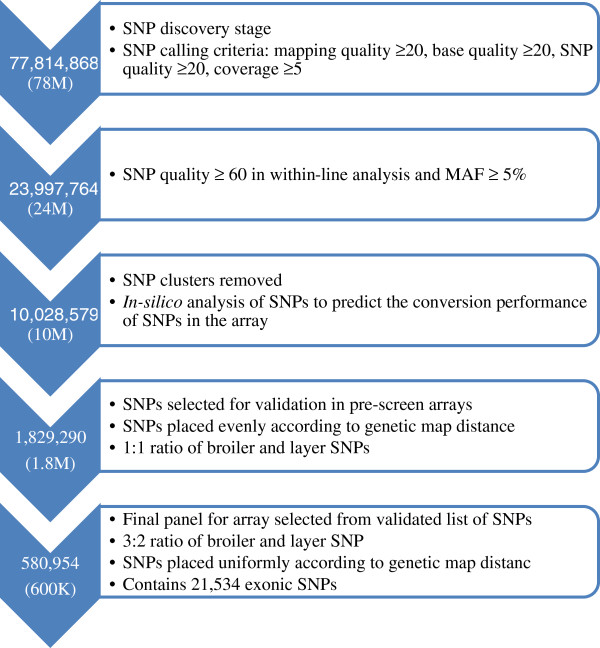
Flow diagram showing the SNP selection steps with major criteria.

Due to the very high number of SNPs detected in the previous steps, a multi-step filtering strategy was followed to reduce the number to a robust and tractable subset for HD array design. The filtering was done on the list of 78 M SNPs obtained from the within-line analyses as the goal was to use SNPs segregating within lines primarily to design an array of most practical use. The 139 M SNP list, however, was consulted for regions where few or no high quality SNPs were detected from within-line analysis. The filtering steps are summarised in Figure 1.

#### SNP reduction based on quality score and low minor allele frequency

In the first step of filtration, an attempt was made to reduce further the occurrence of false positives from the detection stage. SNPs were accepted only if at least one of the following criteria was satisfied: (i) the SNPs with quality scores ≥ 60 and minor allele frequency (MAF) ≥ 0.05 from the within-line detected set; (ii) SNPs from global analysis meeting the same criteria as in (i), irrespective of their quality scores and MAFs in the within-line analyses; (iii) the SNP was present in previous panels or detected by Rubin *et al.*[2]. Filtration based on these criteria resulted in approximately 24 M SNPs.

The above criteria were ignored for SNPs on Chr16 and ChrZ to maximize the retention of putative SNPs which are potentially linked to the MHC gene complex and sex-linked characters, whilst accepting the risk of having some sequencing artefacts.

#### SNP reduction based on close proximity and possible clustering artefacts

In this step, SNPs that were located very close to each other were removed. Very tightly spaced SNPs are less likely to be successfully assayed during genotyping, due to the interference from the neighbouring variant. Also SNP clustering may appear due to mis-alignment of reads due to the presence of InDels (insertions or deletions) at the beginning or end of reads [18]. In developing a 60 K Illumina array for chicken Groenen *et al.*[6] removed SNPs which were in 10 bp of each other on either side but here we retained SNPs that had at least 10 bp of SNP free region on one side and ≥4 bp on the other side because of flexibility of the Affymetrix® Axiom® platform. Again, for Chr16 and ChrZ, all the SNPs were retained without any selection at this stage. The outcome of this filtering was a list of approximately 10 M SNPs.

**Figure 2:**
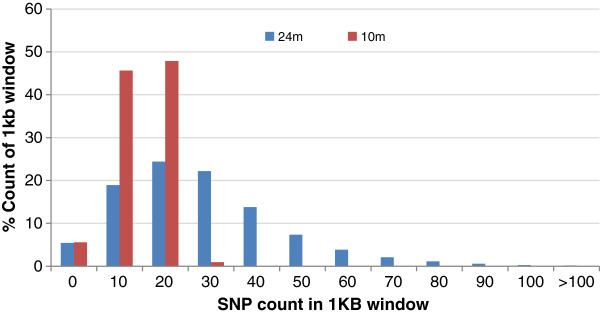
Comparison of SNP density in 1 kb non-overlapping window between 24 M and 10 M lists.

Figure 2 compares SNP density in 1 kb non-overlapping windows between 24 M and 10 M lists. The SNP density varied between 0–207 SNPs per kb in 24 M list with an average of ~24 SNPs/kb and between 0–188 SNPs per kb with an average of ~10 SNPs/kb. The figure shows that about 5% of windows did not have any SNPs after applying the first filtration criteria of SNP quality ≥60, which generated the 24 M list. While the SNP density was quite variable in 24 M list, the 10 M list provides a much more uniform distribution, due to the removal of groups with very closely linked SNPs.

**Figure 3:**
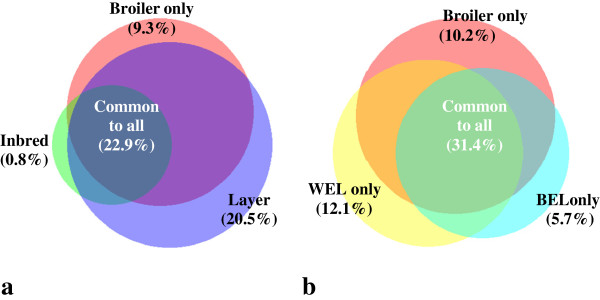
**Venn diagrams showing the share of SNPs in 10 M list among different groups. (a)** Among broiler, layer and inbred lines; **(b)** among broiler, white egg layer (WEL) and brown egg layer (BEL). The size of the circles reflects the relative number of SNPs detected from each groups. (Diagrams created by BioVenn - http://www.cmbi.ru.nl/cdd/biovenn/).

A substantial proportion of the SNPs were shared amongst the different lines, indicating their potential utility to multiple lines, although those spanning across most of the lines are likely to be ancient variants. Venn diagrams in Figure 3 a and b show the shared SNPs between broiler, layer and inbred lines, and broiler, WEL and BEL respectively. In summary, about 23% of the 10 M SNPs were found to be common among broiler, layer and inbred lines while 31% SNPs were found to be common between broiler, BEL and WEL. The size of the circles in Venn diagrams also represents the relative proportion of the SNPs contributed from different groups in the 10 M list.

#### SNP reduction based on *in-silico* analysis of predicted performance on Axiom® arrays

The 10 M SNPs selected in the above step were submitted to Affymetrix for an *in-silico* analysis to predict their reproducibility in the Axiom® array. Two types of design scores were generated from this analysis: (1) *16-mer count,* which is the number of times all 16 bp sequences in the 30 bp flanking region from either side of the SNP had a matched sequence in the genome and (2) *p-convert value,* which arises from a random forest model intended to predict the probability that the SNP will convert on the array. The model considers factors including probe sequence, binding energies, and the expected degree of non-specific binding and hybridization to multiple genomic regions. These scores were generated both for forward and reverse probes. Lower 16-mer counts and greater p-convert values are better for array design. The chance of a SNP converting with a 16-mer count above 100 is extremely low and the chance of conversion increases monotonically as the score decreases. Therefore the thresholds were set to be: 16-mer count ≤ 100 and the p-convert value ≥ 0.2.

**Figure 4:**
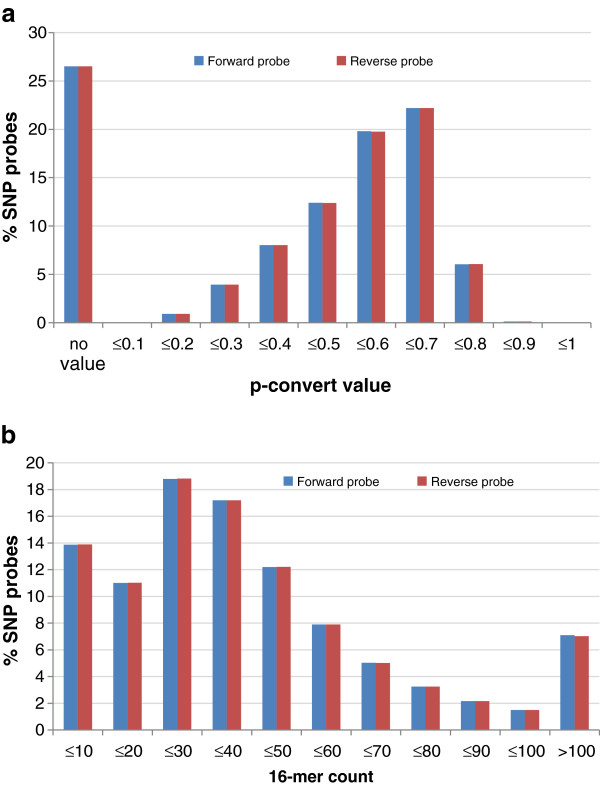
**Distribution of SNP probes in 10 M list based on design scores from Affymetrix *in-silico *analyses. (a)** Distribution based on p-convert values; **(b)** distribution based on 16-mer count.

Of the 10 M SNPs tested, ~73% had a p-convert value ≥ 0.2 and ~93% had a 16-mer count ≤100 (Figure 4 a and b). Taking both the design matrices together, ~66% of the SNPs had the required design scores (i.e. 16 mer count ≤100 and p-convert value ≥ 0.2) irrespective of whether the forward or reverse probes were considered.

#### Selection of SNPs for validation

The fourth step was to prepare a list of ~1.8 M SNPs for validation through genotyping. The objective was to have an even distribution of segregating SNPs across the genome. To achieve this we spread out the SNPs evenly in terms of genetic map distance as provided by Groenen *et al.*[19] instead of using the physical distance, to account for the difference in the cM/Mb ratio between macro- and micro-chromosomes. The ratio of the SNPs segregating within broiler and layer lines was kept at 1:1. A custom made iterative algorithm (see section SNP selection under Methods) was used to select the list of 1.8 M SNPs with the desired criteria. The algorithm first created a backbone by adding evenly spaced SNPs which were segregating across all 24 lines. Then it uniformly placed SNPs for layers that guarantee the target density (in SNPs/cM) for a line. The same step was applied to ensure the target density for broiler SNPs balanced by lines.

### SNP validation result and selection of final 600 K panel

Validation through genotyping of ~1.8 M SNPs was performed by manufacturing three Affymetrix® Axiom® arrays (each array contained ~ 600 K). The major purposes of validation were to assess for each SNP: (1) the conversion performance in the array in terms of genotype call rate, cluster separation and reproducibility, (2) polymorphism in different sets of commercial and wild chicken breeds, (3) stable Mendelian inheritance from parents to offspring, and (4) population characteristics in terms of allele frequency distribution, LD and Hardy-Weinberg equilibrium (HWE) probability. The ultimate goal was to use this information to select a robust set of ~600 K SNPs for the final genotyping array. The validation was carried out on an independent set of 282 individuals including 32 trio samples from three broiler lines, four WEL lines, five BEL lines and 26 individuals from a wide range of diverse traditional breeds of chicken (see Additional file 2: Table S2.xlsx for further details on genotyped samples).

Table 2 summarizes the validation results. Over 64% of the 1.8 M SNPs were found to be reproducible and polymorphic with stable Mendelian inheritance. The rest of the SNPs were rejected as they failed in one or more of these criteria.

**Table 2:** Summary of SNP validation result

	**Number**	**Percent**
Number of SNPs selected for validation	1,829,290	
***Marker conversion success***		
SNPs found polymorphic (in one or more lines)	1,187,482	64.91
SNPs declared monomorphic due to low allele count	313,000	17.11
SNPs failed to convert	328,808	17.97
***Mendelian inheritance check***		
Number of trios checked	32	
Number of polymorphic SNPs failed to show stable Mendelian inheritance in one or more trios	10,674	0.58

Population characteristics of the validated SNPs were explored to make informed decisions about selecting the final 600 K panel. Any SNPs that showed an extreme departure (*P* < 0.00001) from the HWE were removed. While SNPs that show departure from HWE can actually be important as they might represent regions under selection, an extreme departure might indicate genotyping errors, presence of copy number variants (CNVs) or lethal recessive mutation and hence were removed.

**Figure 5:**
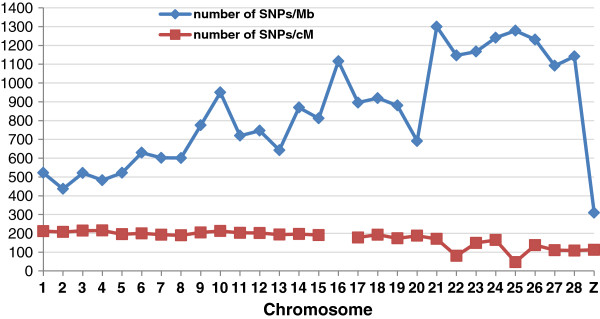
**Chromosome-wise SNP density in the 600 K panel in physical (SNP/Mb) and genetic map distance (SNPs/cM).** The SNPs/cM values were calculated using the genetic map length of each chromosome from Groenen *et al.* (2009).

The same custom algorithm as used in the previous step for selecting 1.8 M SNPs was again used for reducing the 1.8 M to 600 K, with the only difference that the broiler and layer SNPs were selected in a ratio of 3:2. The rationale for choosing a higher proportion of broiler SNPs in the final panel is that a number of recent studies have shown a higher extent of LD in layer compared to broiler [11,13,20] indicating that broilers would require a larger set of segregating SNPs compared to layers to capture the same amount of genetic information. Again the genetic map distance was used to spread the SNPs across the genome. Figure 5 shows the average number of SNPs per unit physical distance and unit genetic map distance, clearly indicating that the SNPs are much more uniformly distributed based on genetic map distance than the physical distance in the final 600 K panel. Since one of the objectives of the panel design was to increase its utility for genome-wide-association studies (GWAS) or GS, it was essential to target equi-distant SNPs across the whole genome. The use of the genetic rather than the physical distance as a unit to distribute SNPs ensured that the density per cM in micro-chromosomes was the same as in macro-chromosomes. The smaller chromosomes have a higher recombination rate and higher gene density [21] and hence, a good coverage is crucial since they may explain a considerable proportion of the genetic variance.

To maximise the utility of the panel for QTL detection studies, all the validated SNPs from exonic or coding regions (n = 21,534) were included in the panel of which 9,345 were predicted to be either non-synonymous or stop-loss/stop-gain types. Table 3 shows the predicted effects of the SNPs that have been included in the final 600 K panel. Studies on human and model organisms predict that most high penetrance (Mendelian or nearly so) diseases or traits are mediated by polymorphisms in the protein coding regions of the genome [22,23]. Although generally the non-synonymous and stop-loss or stop-gain SNPs are implicated to be disease causing, the synonymous SNPs can be useful by being in strong LD with functional mutations. About 15% of the SNPs in our study could not be annotated properly as their co-ordinates could not be resolved unambiguously when mapped to the previous genome assembly (*Gallus_gallus_2.1*) for annotation purpose.

**Table 3:** Summary of annotation of SNPs in 600 K panel to predict the genomic effect

	**Count**	**Percent**
Total number of SNPs in the panel	580,954	
Annotation possible	492,572	84.79
***Annotation result***		
Intergenic	266,636	54.13
Intronic	189,128	38.40
Exonic		
Non-synonymous	9,345	1.90
Synonymous	12,069	2.45
Stopgain/stoploss	120	0.02
1 kb Upstream	5,892	1.20
1 kb Downstream	6,456	1.31
UTR3	2,497	0.51
UTR5	302	0.06
Splicing	83	0.02
Non-coding RNA (ncRNA)	44	0.01

**Figure 6:**
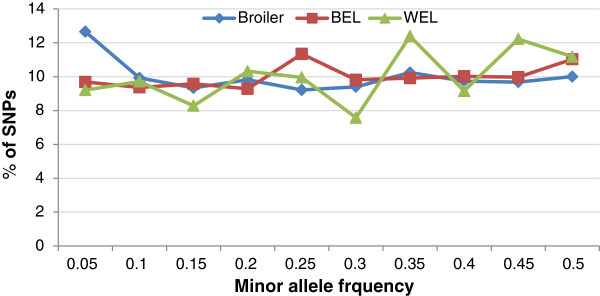
Minor allele frequency distribution of SNPs in the 600 K panel estimated from genotype data.

No direct selection was performed based on MAF as the frequency estimates were not sufficiently precise due to small sample sizes. However, an attempt was made to include SNPs which are common to many lines to maximise the utility of the HD array. Figure 6 shows that the frequency distribution of SNPs is more or less even across different MAF classes across broiler, WEL and BEL groups.

The final 600 K array has 580,954 SNPs in total. Based on the genotyped samples used for validation, the number of polymorphic SNPs ranged between 127,958 to 473,077 per commercial line. This number is expected to rise for each line with screening of more samples. About 21%-53% of the SNPs in the final panel were also found to be polymorphic in the different traditional breeds of chicken that were included in the validation sample set even though only 1 or 2 individuals per breed were analysed. This confirms the versatility of the panel. The final panel includes 1,135 SNPs which were detected on contigs that were assembled *denovo* from unmapped reads in the current study. As mentioned earlier we used the pre-published version of the *Gallus_gallus_4.0* for mapping our reads as this assembly was unpublished at the time of the work. However, recently the assembly has been published in the NCBI database and contains added features such as “unlocalized scaffolds” (for each chromosome these appear as Chr_random) and “unplaced” (Chr_Un). We mapped the *denovo* SNPs against this published reference. This mapping result showed that except 53 SNPs, the rest mapped to either some chromosomes (n = 968 SNPs) or unlocalized scaffolds (n = 22 SNPs) or Chr_Un (n = 92 SNPs). All the SNPs in the panel have been submitted to NCBI’s dbSNP and can be accessed via the link: http://www.ncbi.nlm.nih.gov/projects/SNP/snp_viewBatch.cgi?sbid=1057286.

**Figure 7:**
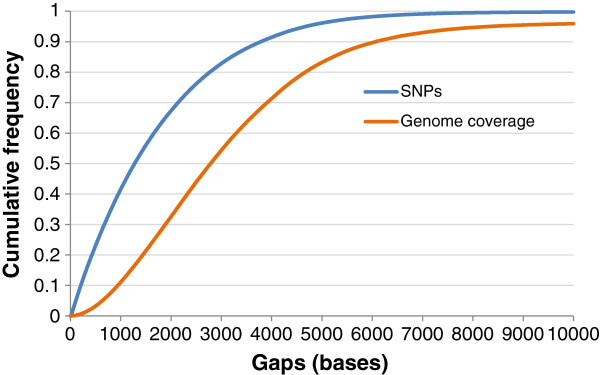
**Cumulative frequency distributions of SNPs and genome coverage as functions of inter-marker spacing in the panel.** Inter-marker spacing included distances between consecutive SNPs and the distances from chromosome ends to the nearest SNP in 600 K panel.

The SNPs in the final array are distributed across the genome with a mean (± SD) inter-marker spacing of 1748 ± 5274 bases. About 98% of the markers are distributed across the genome with a gap size less than 6 kb, but there are some regions which have larger gap sizes and they cover about 10% of the genome (Figure 7). Most of these larger gaps are found in the macro-chromosomes, particularly on Chromosomes 1, 2 and Z.

### PCA analysis of population structure using 600 K array genotypes

**Figure 8:**
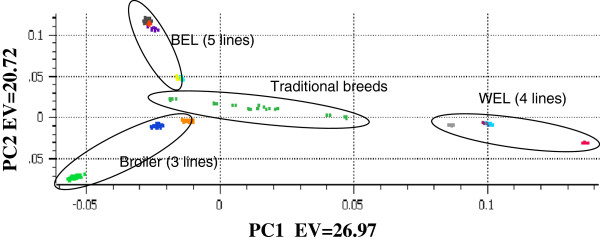
Principal component analysis to investigate the population structure in validated samples using 600 K marker panel.

Detecting the underlying population structure is of interest for many population related studies, particularly for GWAS as population stratification can lead to many spurious associations if not accounted for properly [24]. We performed a principal component analysis (PCA) using the genotype data to investigate the ability of the 600 K panel to detect population stratification in the validated samples. Figure 8 shows the relative co-ordinates of individuals when plotted using the two largest principal components. Individuals originating from the same line clustered together tightly and related groups (e.g. all broiler lines or WEL lines or BEL lines) also appeared in close proximity. The broiler lines were placed relatively close to BEL compared to WEL, while the latter two groups were placed further apart indicating their different origins.

The relatively close proximity of broilers and BELs in the PCA plot is related to their shared ancestry as these breeds were originally established by crossing the European and Asian breeds [25-27]. In contrast, the WEL originated from the single comb White Leghorn breed which is of European origin [25-27]. Our PCA results are thus in agreement with the existing knowledge of the origin of the lines/breeds and are also in agreement with the previous studies such as by Eding *et al.*[28] and Elferink *et al.*[27] which showed phylogenetic relationship among different poultry breeds.

## Conclusions

In this paper we described the development of the first HD SNP array containing 580,954 SNPs for chicken that is more dense compared to the HD arrays available for other farm species such as the 778 K Illumina High-Density Bovine BeadChip Array or the 689 K Affymetrix® Axiom® Genome-Wide BOS 1 Array for cattle considering the fact that the chicken genome is about one third the size of the bovine genome. The array is expected to be useful for GWAS, GS, fine mapping of QTLs, analysis of genome wide selection signatures, detection of CNVs and other genomic analyses and will be available for public and private use.

Important features of our array development approach included: (1) sequencing of pooled samples using a NGS method which has allowed screening of a large number of individuals rapidly and cost effectively leading to the detection of a large collection of SNPs segregating between and most importantly, within diverse populations; (2) applying stringent filtering criteria to minimize inclusion of false positives that appear as artifacts of NGS; (3) inclusion of validated SNPs in the final array that are proven to work in the Axiom® platform; (4) distributing the SNPs on the basis of map distance rather than physical distance to increase the ability to track genomic segments across generations; (5) using an iterative algorithm to select the SNPs to obtain a balanced representation from all the lines tested. The call rates from genotyping using the array are expected to be very high which is confirmed from the initial results of large-scale genotyping in broiler and layer lines (results not shown). We have also shown through validation that the panel will be usable on traditional chicken breeds, different *Gallus* subspecies. The array has been commercially released for academic and industrial use.

Even though we have applied a very thorough approach in developing this array, future projects with a similar goal can consider a number of areas for improvement. One potential area of improvement is barcoding the individual samples before sequencing the pooled DNA as this would enable more information to be extracted from sequence data such as would allow better estimates of the allele frequencies. While this approach was not used in the current project to keep the cost down, future projects may consider this option as the cost of barcoding and library preparation are declining. Moreover, with the rapidly declining cost of sequencing, it would also be possible to increase the coverage and the number of birds for sequencing in future studies. Another area where the future arrays can attempt to improve is the inclusion of more SNPs from Chr16, ChrW and some of the micro-chromosomes, which was not possible in our case due to poor representation of these chromosomes in the current genome build.

## Methods

### Whole genome re-sequencing of chicken lines

The 243 sequenced samples used for SNP detection were supplied by Aviagen (broilers), Hy-line International and Lohmann of Synbreed Consortium (white and brown egg layers), the Pirbright Institute (formerly known as Institute for Animal Health) (inbred lines) and the Roslin Institute (RI-J experimental layer line) (see Table 1). Except for the Synbreed samples, sequencing for all other lines was performed by the ARK Genomics lab at the Roslin Institute, UK. The Synbreed samples were sequenced by Helmholtz Zentrum München, Germany.

DNA was extracted from blood samples. For each line equal quantities of DNA from 10–15 individuals were mixed to produce pooled samples, except for one line for which three samples were sequenced individually (see Table 1). The sequencing libraries were prepared using the Illumina genomic DNA sample prep kit according to the protocol, “Preparing Samples for Sequencing Genomic DNA rev B (Illumina)”. Sequencing was performed on Illumina GAIIx platform using a paired end protocol. For Synbreed samples the length of paired reads was 76 bases while for the rest of samples it was 101 bases.

### SNP detection

SNP detection was done by aligning the Illumina sequence reads to the chicken reference genome (*Gallus_gallus_4.0*, pre-published version) obtained from Washington University [15].

Alignment was performed for paired end reads using Burrows-Wheeler Aligner (BWA) v0.5.7 [29] using default setting. Prior to mapping, the repeat regions in the reference genome were masked using RepeatMasker [30]. To further remove other areas of the genome with potential problems, 16-mers occurring more than 5 times were also masked, although this had negligible effect on the genome as the majority of such sequences had already been masked using RepeatMasker. SAMtools v0.1.7 [31] was used to remove potential PCR duplicates among the sequence reads and to call SNPs. Only reads that mapped to single unique position on the genome were used. SNP detection was done in two stages. Firstly, each line was analysed separately to identify SNPs segregating within the line followed by a combined analyses of all the lines to detect more SNPs from low depth-of-coverage regions.

Some of the sequence reads failed to map to the reference genome. These were assembled *de-novo* into contigs using the package SOAPdenovo [32] for broilers and layers separately. The kmer size was set as 31.

### SNP selection

SNP selection was done in multiple steps using several criteria. The filtration criteria were set with the aims to minimize the risk of choosing false positives, select SNPs with an even distribution across the genome in terms of cM distance, and obtain a balanced representation of broiler and layer SNPs. In order to achieve an even distribution of SNPs segregating in different lines, a custom-made algorithm was applied which is described below. To facilitate an efficient querying of the SNP data, a NoSQL database (in MongoDB) was created.

An iterative divide-and-conquer algorithm was developed to select efficiently both the pre-screening 1.8 M panel and the final 600 K panel. Although the SNP selection was performed on each chromosome separately, the number of combinations of candidate panels was overwhelming even for the smallest chromosomes. To simplify and accelerate the process, the SNP selection was performed between “backbone” SNPs, which were common between lines. The implication was that the regions between backbone SNPs were far smaller and hence the number of candidate SNPs was dramatically smaller. The algorithm consisted of two major steps, the construction of the backbone panels and the filling of the gaps for each line.

The first step was to populate the baseline backbone panel by including the *N* SNPs that segregated between all lines and were more than *t* bases apart (*t* was a threshold to maintain minimum distance between SNPs; t = 2 kb corresponding to the approximate expected density of a 600 K panel on a 1Gb long genome). This resulted in having *N + 1* segments instead of the whole chromosome to fill. A second backbone was constructed consisting of *I* SNPs segregating in the inbred lines, which ensured that for these non-commercial lines there were *N + I* SNPs already in the candidate panel that were spread across the chromosomes providing a good coverage without big gaps. There were also *N + (I-I*_*u*_*)* SNPs*,* (where *I*_*u*_ the number of SNPs unique to inbred lines), that were also segregating in at least one of the commercial lines. A third backbone was constructed, containing *L* SNPs that were segregating in all layers to fill the *N + (I-I*_*u*_*) + 1* gaps. The next backbone included *B* SNPs that were present in all broiler lines, and filled all the *N + (I-I*_*u*_*) + (L-L*_*u*_*) + 1* segments, where *L*_*u*_ are the SNPs for *L* that do not segregate in any broiler lines. The final backbone consisted of *X = N + I + L + B* SNPs. For the final selection from 1.8 M to 600 K, where information about coding SNPs was available, all *C* SNPs in coding regions that were not already in the backbone were added to the backbone.

The second step was to start filling the regions for each line separately. For the *i*^*th*^ line, the gaps to evenly distribute SNPs were equal to *X-F*_*i*_ *+ 1*, where F_i_ were the SNPs not segregating in the line of interest. Iteratively, every segment was filled by *n* SNPs, where *n* was a function of the target density for the i^th^ line and the length of the segment. For the pre-screening panel the same density was used for all lines, but for the final panel, broilers had a higher density due to their shorter intra-marker LD. By calculating the *n* SNPs that had to be evenly distributed in the segment, the algorithm chose the SNPs that were closer to the ideal positions by minimising the sum of squared distances between consecutive SNPs in order to approximate the target density. If the gap was too small, meaning less than the target density, no SNP was placed.

In total S_i_ SNPs were placed for the i^th^ line and the backbone was updated, now containing X + *S*_*i*_*-F*_*(i+1)*_ *+ 1* for the next line *i + 1*, where *F*_*(i+1)*_ was the number of backbone SNPs not present in the *i + 1*^th^ line. We started with the white layers, followed by the brown and finished with the broiler lines, following the order in the extent of LD.

The advantage of this strategy was to avoid having SNPs segregating in more than one line that were very closely located, which in turn meant a more even distribution was achieved in all lines and thus maximising the utility of the SNPs in the panel. No selection for MAF was enforced, as the estimates were biased, because either those were estimated solely on sequencing data on pools with variable depth coverage, or were available only for the broiler lines with sufficient samples in the pre-screening phase.

### SNP validation

About 1.8 M selected SNPs were validated on 282 individuals (see Additional file 2: Table S2.xlsx for further details). DNA samples of commercial birds were supplied by Aviagen, Hy-line International and Lohmann and the samples of different traditional chicken breeds were taken from a DNA collection established during the EC project AVIANDIV and follow up studies as described elsewhere [33,34].

Genotyping was done on three Axiom® arrays using the Affymetrix® GeneTitan® system according to the procedure described by Affymetrix (http://media.affymetrix.com/support/downloads/manuals/axiom_genotyping_solution_analysis_guide.pdf). The population characteristics of the SNPs such as allele frequency, HWE probability based on Fisher’s Exact Test and LD between adjacent pair of SNPs were explored using the software SNP & Variation Suite (SVS version7) of Golden Helix Inc. The genomic positions of the SNPs and their effect on protein coding were predicted by annotating them against the Ensembl gene annotation database for chicken based on *Gallus_gallus_2.1* chicken reference genome. The software package ANNOVAR [35] was used for this purpose.

### Use of 600 K array for studying population structure

A PCA was performed using the genotype data of the autosomal SNPs from the 600 K panel on all the unrelated samples (n = 218) to assess the utility of the panel in detecting population structure. PCA was carried out using SVS (version 7) package of Golden Helix Inc. Each marker’s data was normalized by its theoretical standard deviation under HWE and an additive model was considered.

## Abbreviations

BEL: Brown Egg Layer; CNV: Copy Number Variant; GS: Genomic Selection; GWAS: Genome Wide Association Study; HD: High Density; HWE: Hardy-Weinberg Equilibrium; InDel: Insertion or Deletion; LD: Linkage Disequilibrium; M: Million; MAF: Minor Allele Frequency; NGS: Next Generation Sequencing; PCA: Principal Component Analysis; QTL: Quantitative Trait Loci; SNP: Single Nucleotide Polymorphism; WEL: White Egg Layer.

## Competing interests

Four commercial organisations - Aviagen, Hy-line International, Affymetrix, and Lohmann- were involved in the development of the array and in preparation of the manuscript. Among the authors, AK and KAW work for Aviagen Ltd, JF works for Hy-line International, RP works for Lohmann, AP and FB work for Affymetrix and FB also holds shares in the Affymetrix. This project was funded by DEFRA/BBSRC LINK grant promoting industry-academia collaboration.

## Authors’ contributions

The work presented here was carried out in collaboration between all authors. DWB, JAW, AK and KAW defined the research programme. AK, AAG, CB, KAW, JAW and DWB designed methods and experiments, analysed the data, interpreted the results and wrote the paper. FT, SS and RT collected and processed the raw sequence data and SNP detection. LY performed the *de novo* assembly of unmapped sequence reads. AP and FW performed the Affymetrix® Axiom® design and validation assays. PK, PMH, MF, NS, JF and KAW provided DNA samples and information on poultry lines. TMS, GH, SW, RP, MG, SQ and HS provided sequence data and DNA samples from the Synbreed consortium. DWB and JAW co-designed experiments, discussed analyses, interpretation, presentation and coordinated the project. All authors have contributed to, seen and approved the manuscript.

## Supplementary Material

Additional file 1: Table S1 An Excel file with the number of SNPs detected from within-line-analyses and selected at various stages across different chromosomes. Click here for file

Additional file 2: Table S2 An Excel file providing description of the genotyped samples used for validation of the 1.8 M SNPs. Click here for file
